# Integrated genomic and transcriptomic Insights into methanol tolerance mechanisms in *Methylobacterium extorquens* AM1, identifying key targets for strain engineering

**DOI:** 10.1186/s13036-025-00557-1

**Published:** 2025-12-08

**Authors:** Gyu Min Lee, Khoi Nhat Pham, Ina Bang, Seyoung Ko, Donghyuk Kim

**Affiliations:** School of Energy and Chemical Engineering, Ulsan National Institute of Science and Technology (UNIST), Ulsan, 44919 Republic of Korea

**Keywords:** Methylotrophy, Methanol, Carbon neutrality, Adaptive laboratory evolution, Metabolic engineering, Systems biology, Methylobacterium extorquens

## Abstract

Methanol is an attractive one-carbon feedstock for sustainable biomanufacturing because of its abundance, cost-effectiveness, and industrial compatibility. However, its cytotoxicity limits its biotechnological applications in native methylotrophs such as *Methylobacterium extorquens* AM1. In this study, we developed AM1-derived strains capable of sustained growth under elevated methanol concentrations through adaptive laboratory evolution (ALE). From the evolved population, five representative strains were isolated, exhibiting up to a 1.68-fold increase in specific growth rates compared with those of the wild- type at 2.5% (v/v; 617.93 mM) methanol. Genomic analysis of the evolved strains revealed recurrent mutations in *metY* (*O*-acetyl-L-homoserine sulfhydrylase) and *kefB* (potassium efflux antiporter). Functional validation confirmed that these recurrent mutations improve methanol tolerance through distinct yet complementary mechanisms. The consistent emergence of mutations in *metY* and *kefB* across all strains implies strong convergent selection, highlighting their independent roles in a coordinated adaptive strategy. Specifically, the *metY* mutations are hypothesized to fine-tune enzyme activity to reduce toxic byproduct formation, while the loss-of-function *kefB* mutation likely conserves cellular energy. The largely additive nature of their combined effect underscores how these distinct adaptive mechanisms, optimization of methionine biosynthesis and energy conservation, independently contribute to the overall fitness improvement under methanol stress. To further elucidate methanol adaptation strategies, we performed an integrated genomic and transcriptomic analysis. Transcriptome profiling revealed 767 differentially expressed genes, indicating widespread transcriptional reprogramming. Notably, the key upregulated genes were involved mainly in central carbon metabolism, methionine biosynthesis, cellular defense responses such as oxidative stress mitigation, and nitrogen metabolism, as interpreted through DEG mapping onto metabolic pathways using a genome-scale metabolic model. Overall, this study highlights how coordinated genetic and transcriptional adaptations contribute to methanol tolerance in the AM1-derived evolved strains, providing systems-level insights. These strains represent promising platforms for methanol-based biomanufacturing, with the potential to improve microbial robustness and reduce stress-induced bottlenecks in industrial processes.

**Supplementary Information:**

The online version contains supplementary material available at 10.1186/s13036-025-00557-1.

## Background

Methanol is an abundant and cost-effective one-carbon (C1) feedstock compatible with existing bioprocessing infrastructure, making it a promising substrate for sustainable biomanufacturing [1, 2]. Its increasing availability from both natural gas and renewable sources, combined with decreasing production costs, further supports its industrial adoption [3–7]. The chemical versatility of methanol enables its conversion into diverse high-value products such as organic acids and biopolymers, reinforcing its potential role as part of circular bioeconomic strategies to reduce carbon emissions [8–10].

Methanol is toxic through both direct membrane disruption and indirect metabolic effects, including oxidative stress, cytoplasmic acidification, and the accumulation of *O*-methyl-L-homoserine (methoxine), a toxic analog of L-methionine that exacerbates cellular damage [11–17]. This analog is erroneously incorporated into elongating peptides, leading to translational errors and cellular toxicity [13]. These multifaceted toxic effects necessitate a comprehensive understanding of microbial adaptation mechanisms to methanol stress. Such insights are critical not only for elucidating methanol-induced stress response strategies but also for enabling robust microbial platforms in methanol-based bioprocesses.

*Methylobacterium extorquens* AM1 is a model facultative methylotroph with well-characterized methylotrophic pathways. Methanol-based bioproduction has been extensively studied for the synthesis of diverse value-added compounds, such as amino acids, organic acids (e.g., succinic acid), and biopolymers (e.g., polyhydroxyalkanoates) [18–23]. However, the industrial application of *M. extorquens* AM1 is hindered by its low methanol tolerance. Growth inhibition occurs at methanol concentrations exceeding 1% (v/v; 247.18 mM), with complete inhibition observed at 5% (v/v; 1.24 M) [24]. As previous studies have reported, this limitation forces the use of suboptimal methanol concentrations (~ 0.5% v/v; ~ 123.59 mM) in bioprocesses, thereby reducing economic viability [25–29].

To address these challenges, adaptive laboratory evolution (ALE) has emerged as a powerful tool for improving microbial tolerance to environmental stresses through the selection of beneficial phenotypic traits under controlled conditions [30–32]. Unlike rational genetic engineering approaches that require prior knowledge of specific targets, ALE enables the discovery of novel adaptive mechanisms through continuous selection for beneficial mutations and regulatory rewiring. Previous studies have successfully applied ALE to enhance methanol tolerance in *M. extorquens* AM1, identifying beneficial genetic mutations under elevated methanol concentrations [33, 34].

While these studies have substantially advanced our understanding of methanol tolerance, most have focused primarily on identifying adaptive mutations, with comparatively limited emphasis on how such mutations influence cellular systems at the transcriptional and functional levels. A deeper and integrative understanding of these relationships is essential for elucidating the mechanisms by which methanol tolerance arises and for informing future strain engineering efforts [30, 35].

To bridge this gap at a systems level, genome-scale metabolic models (GEMs) offer a powerful predictive framework. These models are widely used to simulate complex metabolic responses, such as optimizing the utilization of diverse carbon sources from C1 gases to complex aromatics like phenolics, and designing metabolic engineering strategies for producing chemicals like ethanol [36–38]. Beyond these dynamic applications, GEMs are also critical for defining the fundamental structural properties of a metabolic network. A primary example of this foundational analysis is the prediction of gene essentiality, which identifies the core metabolic functions indispensable for cell survival under a given condition. In this context, the GEM of *M. extorquens* AM1 was utilized to establish this baseline understanding [19], providing an in silico scaffold to interpret experimental data and identify critical targets for subsequent strain engineering.

In this study, a multilayered systems approach was employed to investigate the adaptive strategies of *M. extorquens* AM1 strains evolved for sustained growth at elevated methanol concentrations. This approach integrates genome resequencing, transcriptomic profiling, and both GEM- and structure-guided interpretation, culminating in the functional validation of key recurrent mutations. The aim is to provide mechanistic insight into how specific mutations reshape metabolic and stress response networks to support methanol tolerance. These insights are intended to lay the foundation for engineering efficient and resilient microbial platforms for methanol-based biorefineries, enabling higher substrate loadings and mitigating stress-associated growth limitations in industrial applications.

## Materials and methods

### Bacterial strains and culture conditions

All strains and plasmids used in this study are listed in Table 1. The wild-type *M. extorquens* AM1 strain was obtained from a laboratory glycerol stock and used as the parental strain for all subsequent experiments. *M. extorquens* AM1 and its evolved derivatives were grown in modified minimal medium containing methanol as the sole carbon source. The base composition of the minimal medium followed a previously described formulation, which notably included molybdenum but lacked tungstate [41]. To enhance methanol metabolism, two modifications were introduced. The minimal medium was supplemented with 30 μM of disodium tungstate dihydrate (Na_2_WO_4_·2H_2_O; 9.90 mg/L) based on its essential roles in the ethylmalonyl-CoA (EMC) pathway and formate oxidation [42]. Additionally, the final concentration of cobalt was increased to 12.0 µM by adding cobalt(II) chloride hexahydrate (CoCl_2_·6H_2_O; 2.86 mg/L) [43]. All cultures were incubated at 30 °C with shaking at 200 rpm. Rifamycin and kanamycin were added at 50 µg/ml when appropriate. All reagents were purchased from Sigma-Aldrich.

**Table 1:** Bacterial strains and plasmids used in this study

Category	Strain/Plasmid	Description	Source
*E. coli* strains	DH5α	F’ Φ80lacZ• ΔM15 •ƒ(lacZYAargF) U169 deoR recA1 endA1 hsdR17(rk-, mk +) phoA supE44 thi-1 gyrA96 relA1	enzynomics
	DH5α_mT1	DH5α-derivate with pQSAK-tet::mT1 plasmid	This work
	DH5α_mT2	DH5α-derivate with pQSAK-tet::mT2 plasmid	This work
	DH5α_kKO	DH5α-derivate with pQSAK-tet::kKO_loxP plasmid	This work
*M. extorquens* AM1 strains	WT	Wild-type strain	Laboratory stock
	Am01	Single strain isolated from cell population after adaptive laboratory evolution (ALE)	This work
	Am02	Single strain isolated from cell population after ALE	This work
	Am03	Single strain isolated from cell population after ALE	This work
	Am04	Single strain isolated from cell population after ALE	This work
	Am05	Single strain isolated from cell population after ALE	This work
	mT1	Single mutant strain having SNP F36L in *metY* (MEXAM1_RS11880) gene	This work
	mT2	Single mutant strain having SNP S383L in *metY* gene	This work
	kKO	Single mutant strain knocked out *kefB* (MEXAM1_RS12770) gene	This work
	kKO + mT1	Double mutant strain having SNP F36L in *metY* gene and knocked out *kefB* gene	This work
	kKO + mT2	Double mutant strain having SNP S383L in* metY *gene and knocked out *kefB* gene	This work
Plasmids	pQSAK	Backbone plasmid for the construction of pQSAK-tet; catR, KanR, ampR	[39]
	pQSAK-tet	Backbone shuttle vector designed to deliver SNP sequences to *M. extorquens* AM1 and facilitate knock-out experiments; catR, tetR, ampR	This work
	pCM184	Plasmid containing a loxP-kanamycin resistance cassette; AmpR, KanR, tetR	[40]
	pQSAK-tet::mT1	Fragment containing the mutated *metY* region of strain Am01, cloned into the pQSAK-tet shuttle vector	This work
	pQSAK-tet::mT2	Fragment containing the mutated *metY* region of strain Am02, cloned into the pQSAK-tet shuttle vector	This work
	pQSAK-tet::kKO_loxP	Fragment containing upstream and downstream sequences flanking the *kefB* gene, along with a loxP-kanamycin resistance cassette, cloned into the pQSAK-tet shuttle vector	This work
	pCM157	Plasmid encoding Cre recombinase to excise the remaining loxP site; tetR	[40]

‘Category' column indicates the classification of each entry, such as *E. coli* strains, *M. extorquens* AM1 strains, or Plasmids

‘Strain/Plasmid' column specifies the name of the bacterial strain or plasmid used in the study. This includes wild-type, evolved strains, engineered mutants, and genetic constructs

‘Description' column provides a brief explanation of each strain or plasmid, including genetic modifications (e.g., SNPs, gene knock-outs), source lineage (e.g., ALE-derived strain), or cloning details (e.g., specific insert or resistance marker). Key features such as the presence of selection markers or recombinase sites are also noted

‘Source' column indicates the origin of each entry, specifying whether it was newly constructed in this study (This work), obtained from laboratory stock, or purchased from a commercial supplier (e.g., enzynomics)

### Optimization of cobalt concentration

To determine the optimal cobalt concentration, a growth phenotype assay was performed prior to the ALE experiments using the wild-type strain. These assays were conducted using the base minimal medium, which was supplemented with 30 µM disodium tungstate dihydrate. A wide range of cobalt concentrations was selected and tested to systematically investigate the dose–response relationship of cobalt on methanol metabolism. The standard concentration (1.2 µM) served as the baseline. A concentration below the standard (0.6 µM) was chosen to test for growth limitation under the baseline condition. To explore the optimal range for growth enhancement, concentrations significantly above the baseline (6.0 and 12.0 µM) were selected, representing approximately a log-scale increase. Finally, a high concentration (120.0 µM) was included to identify the saturation point of the growth response or any potential inhibitory effects. Cultures were inoculated in duplicate at an initial OD_600_ of approximately 0.01, with OD_600_ measurements taken every 6 h to assess growth performance. The specific growth rates were subsequently calculated from these measurements using GrowthRates v.6.2 [44].

### Adaptive laboratory evolution (ALE)

ALE was performed to generate an evolved AM1 population with enhanced methanol tolerance. The wild-type strain from the glycerol stock was initially precultured in 50 mL of modified minimal medium containing 0.5% (v/v) methanol in a 250 mL baffle flask until it reached an optical density at 600 nm (OD_600_) of approximately 3.40. The culture was then serially transferred to fresh medium, starting at an initial OD_600_ of approximately 0.05.

Following an initial adaptation period at 0.5% methanol, the concentration was gradually increased in 0.25% increments during the ALE process. Each increase was implemented only after consistent growth improvement was observed in two consecutive transfer cultures, as determined by the higher OD_600_ measured at a fixed time point (typically ~ 20–22 h) than that of the previous culture. This empirical criterion ensured that methanol concentrations increased only to populations demonstrating reliable growth improvement, thereby minimizing the risk of growth collapse during ALE.

The adaptation process continued until stable growth was achieved with 2.5% methanol, which corresponds to approximately 796 generations. After the ALE process was complete, the evolved cell populations were spread on agar plates containing modified minimal medium supplemented with methanol and incubated at 30 °C. Five evolved strains (Am01-Am05) were selected and stored in 25% glycerol at −80 °C.

### Growth phenotype assays of evolved and engineered strains

The methanol tolerance of the evolved and engineered strains was characterized through growth phenotype assays. These experiments involved two distinct groups of strains. The first group comprised the wild-type strain and five evolved strains (Am01, Am02, Am03, Am04, and Am05), whereas the second group consisted of the wild-type strain along with engineered strains targeting *metY* and *kefB*. These included single mutants (mT1, mT2, kKO) and double mutants (kKO + mT1, kKO + mT2).

All strains were first precultured in 50 mL of modified minimal medium in 250 mL baffle flasks containing 0.5% methanol at 30 °C and 200 rpm. The cultures were subsequently diluted to an initial OD_600_ of approximately 0.02 and transferred into 50 mL of fresh modified minimal medium containing either 0.5% or 2.5% methanol. For the first group (evolved strains), OD_600_ was measured every 3 h until the stationary phase; for the second group (engineered strains), measurements were taken every 4 h. All experiments were performed in duplicate. Growth parameters were calculated for each biological replicate, and the average and standard deviation of these parameters are reported.

The growth parameters were modeled using GrowthRates v.6.2, which calculates maximum growth rates (μmax), doubling times, and lengths of the lag phase [44]. The value of μmax was determined by plotting ln (OD_600_) versus time and identifying the maximum slope of a best-fit trend line incorporating at least five data points.

For engineered strains carrying combined double mutations, additive growth effects were assessed by calculating the expected growth rate improvement (Δμ) as the sum of the individual Δμ values relative to those of the wild-type. The deviation between the observed and expected Δμ (denoted as ε, epistasis) was computed to evaluate epistatic interactions, with positive ε indicating synergy and negative ε indicating antagonism. These values were derived from the μmax estimates.

### Whole-genome resequencing, structural modeling, and pan-genome analysis

Genomic DNA was extracted from the five evolved strains (Am01-Am05) using the Qiagen MagAttract HMW DNA Kit. DNA libraries were prepared using the TruSeq DNA PCR-Free Kit and sequenced at DNA Link, Inc. (South Korea) on the Illumina NovaSeq 6000 platform (2 × 151 bp) with an insert size of 550 bp.

Mutations were identified using breseq v.0.36.1 in consensus mode with the default parameters [45]. The gdtools utility of breseq was used to incorporate the predicted mutations and generate a FASTA file for the evolved strains. To distinguish ALE-induced mutations, the wild-type *M. extorquens* AM1 genome was also resequenced and compared against the reference genome (GCF_000022685.1). Mutations not introduced during the ALE process were excluded from further analysis. Specifically, six synonymous mutations that did not alter protein sequences and eight mutations shared by both the wild-type and evolved strains were filtered out. Functional domain analysis of the *kefB* gene was performed using InterProScan [46].

The protein structure of MetY from *M. extorquens* AM1 was predicted using AlphaFold3 [47]. The reliability of the predicted structure was evaluated using the per-residue confidence score, pLDDT (predicted Local Distance Difference Test), which AlphaFold3 stores in the B-factor column of the output PDB file. Structural homologs of MetY were identified using the Foldseek Search Server, and structural similarity between MetY and selected homologs was quantified by calculating template modeling (TM) scores using the TM-align server [48, 49]. All subsequent structural analyses were performed using PyMOL v.3.1.4 [50]. The model was colored by its pLDDT values to visually assess local prediction quality. To further assess structural similarity, the predicted AM1 MetY structure was superposed with the experimentally determined MetY structure from *Thermotoga maritima* (PDB: 7KB1) [51]. After structural alignment, the distances between the PLP ligand and the mutated residues were measured using the ‘distance’ function to evaluate their potential influence on enzyme function.

Pan-genome analysis was conducted on 13 *M. extorquens* strains, including the five evolved strains. The dataset consisted of the following strains: *M. extorquens* AM1 (GCF_000022685.1), *M. extorquens* ATCC 55366 (GCF_030062705.1), *M. extorquens* CM4 (GCF_000021845.1), *M. extorquens* DM4 (GCF_000083545.1), *M. extorquens* NBC_00036 (GCF_026122615.1), *M. extorquens* NBC_00404 (GCF_026122595.1), *M. extorquens* PA1 (GCF_000018845.1), *M. extorquens* PSBB040 (GCF_001971665.1) and the five evolved strains (Am01-Am05). The latest RefSeq annotations for eight of these strains (*M. extorquens* AM1, *M. extorquens* CM4, *M. extorquens* DM4, *M. extorquens* NBC_00036, *M. extorquens* NBC_00404, and *M. extorquens* PSBB040) were retrieved from the public database of NCBI in RefSeq format, using GBFF files of the ‘complete genome’ assembly level.

The RefSeq annotation for the *M. extorquens* TK 0001 strain was previously available but was later removed from the NCBI database because of the presence of numerous frameshifted proteins. Consequently, it was excluded from the pan-genome analysis. Bacterial Pan Genome Analysis (BPGA) was used to construct a neighbor-joining phylogenetic tree and identify ortholog clusters with a protein sequence identity cutoff of 90% [52]. Since the BPGA operates exclusively on protein sequences, pseudogenes were excluded from the analysis. A pan-genome curve was generated to determine whether the collective genomes of *M. extorquens* follow an open or closed model.

Notably, genes shared only among the evolved strains and the wild-type were initially classified as accessory genes because of their presence in multiple strains but were reclassified as unique, as they were found exclusively in AM1-derived genomes.

### Genetic modifications and functional validation

Genetic modifications were performed in the wild-type *M. extorquens* AM1 background to confirm the causal relationship between the recurrently observed mutations in *metY* and *kefB* and the methanol-tolerant phenotype. These modifications were introduced through homologous recombination using the pQSAK-tet suicide vector [39, 40]. This reverse genetics approach is essential for confirming that these specific genomic changes are indeed responsible for adaptive traits rather than random, nonadaptive mutations. This vector, modified from pQSAK by replacing the kanamycin resistance gene with a tetracycline resistance gene, was linearized by PCR using PrimeSTAR® GXL DNA Polymerase (TaKaRa Bio, Japan) and assembled with target fragments using NEBuilder HiFi DNA Assembly. All cloning steps were conducted in *E. coli* DH5α (Enzynomics, South Korea), with transformants selected on kanamycin-containing LB agar and verified by colony PCR.

The constructs were introduced into *M. extorquens* AM1 via electroporation using competent cells prepared by glycerol washing. The cells were recovered overnight in minimal medium supplemented with 0.5% methanol and spread onto rifamycin- and kanamycin-supplemented agar plates. After 8 days, colonies were screened for single-crossover integrants. For *kefB* knock-out, *sacB*-based counterselection was used on sucrose-containing media to isolate double-crossover recombinants. Cre-lox recombination was then performed through pCM157 introduction to excise the kanamycin cassette, with excision confirmed by PCR and Sanger sequencing.

For *metY* SNP introduction, point mutations F36L (mT1) and S383L (mT2) were amplified from evolved strain genomic DNA. The fragments were subsequently cloned and inserted into pQSAK-tet and introduced as described above. Owing to the essential role of *metY*, all media used during the integration and selection steps were supplemented with 167.55 µM (25 µg/mL) L-methionine. Marker removal was not required for *metY* SNPs. Merodiploid and counterselected strains were verified by PCR and Sanger sequencing.

Double mutants (kKO + mT1, kKO + mT2) were constructed by introducing each *metY* SNP construct into the *kefB* knock-out background using the same procedure. The final mutants were validated by Sanger sequencing. A detailed list of primer sequences is provided in Table S1, and the stepwise workflow is illustrated in Fig. S1.

### RNA isolation and RNA-seq

To isolate total RNA, 2 mL of mid-exponential phase cells was harvested and stabilized using RNAprotect Bacteria Reagent (Qiagen, USA). The mid-exponential phase for each strain under each condition was defined based on the exponential phases previously determined by GrowthRates analysis. Specifically, cells were harvested when the culture reached a target OD_600_ within the following ranges: 0.7–0.9 for evolved strains and 0.6–0.7 for the wild-type in 0.5% methanol; and 1.8–2.0 for evolved strains and 1.7–1.8 for the wild-type in 2.5% methanol. The harvested cells were then lysed, and total RNA was extracted using the RNeasy Plus Mini Kit (Qiagen, USA).

To remove abundant ribosomal RNA (rRNA), total RNA was treated with the Pan-Prokaryote riboPOOL kit (siTOOLs Biotech, Germany) and Dynabeads™ MyOne™ Streptavidin C1 (Invitrogen, USA). The rRNA-depleted RNA was purified using the RNA Clean & Concentrator Kit (Zymo Research, USA).

RNA fragmentation was performed by shearing, followed by second-strand synthesis to generate cDNA using the KAPA Stranded RNA-Seq Library Preparation Kit (Roche, Switzerland). The prepared Libraries were sequenced using Illumina high-throughput platforms, including the NextSeq 550 and NovaSeq 6000, according to standard protocols.

### Differential gene expression analysis

The quality of the raw sequencing reads was assessed using FastQC v.0.12.1 and preprocessed using Trimmomatic v.0.32 to eliminate low-quality and unpaired reads prior to read alignment [53, 54]. To mitigate the high abundance of rRNA reads, which can interfere with the mRNA transcript alignment, rRNA genes were indexed using STAR aligner v.2.7.10b [55]. Computational rRNA removal was performed by aligning reads to the indexed rRNA reference using STAR aligner. Reads that did not align to rRNA genes were retained for further analysis.

The reference genome of each evolved strain was indexed using STAR aligner. The aligned reads were then mapped to the corresponding evolved strain reference genome, which was generated using breseq. Mapping was performed using STAR aligner with the same parameters as those used for computational rRNA removal. The resulting BAM files were sorted using SAMtools v.1.19.2 and converted to GFF format using Python3 and Pysam v.0.22.1 [56].

Differential gene expression was conducted using DESeq2 v.1.36 [57] in R. Read counts per gene were obtained with the ‘summarizeOverlaps’ function from the ‘GenomicAlignments’ package, following the Bioconductor RNA-Seq workflow [58, 59]. Size factor normalization was applied to account for differences in sequencing depth and library composition, ensuring that gene expression comparisons reflected biological variation rather than technical bias [59]. The resulting ‘SummarizedExperiment’ object, containing read count data, was converted into a ‘DESeqDataSet’ for analysis.

To distinguish adaptive transcriptional responses from general methanol-induced changes, interaction terms were implemented using the DESeq2 design formula (design = ~ strains_group * methanol). In this design, ‘strains_group’ distinguished wild-type and evolved strains, whereas ‘methanol’ indicated the methanol concentration (0.5% vs 2.5%). This approach enabled the identification of DEGs uniquely driven by strain-specific responses to methanol changes. The resulting log_2_ fold changes represented the difference in the methanol response between strain types rather than the main effects of strain or methanol alone.

Genes with significant interaction effects were extracted using the ‘results’ function in DESeq2, with significance thresholds set at adjusted *p*-value (false discovery rate, FDR) < 0.05 and absolute log_2_ fold change > 1. The filtered DEGs, including those identified through interaction effects, were used for downstream analyses, as they capture transcriptional changes specifically associated with adaptive evolution. Global transcriptomic variation across conditions was visualized using principal component analysis (PCA) and volcano plots. Further details on the application of interaction terms can be found in the DESeq2 vignette [57], available at http://bioconductor.org/packages/release/bioc/vignettes/DESeq2/inst/doc/DESeq2.html#interactions.

### Functional classification and enrichment analysis of DEGs

Gene Ontology (GO) enrichment analysis was performed to classify DEGs into functional categories [60]. The GO term database for *M. extorquens* AM1 (“272,630.protein.enrichment.terms.v12.0.txt”) was obtained from the STRING database and filtered to include only terms associated with Biological Process (BP), Cellular Component (CC), and Molecular Function (MF) [61]. The locus tags in this database were originally annotated using GenBank. These annotations were converted to RefSeq annotations, and genes lacking corresponding RefSeq annotations were excluded from the analysis.

Enrichment analysis was conducted separately for the upregulated and downregulated genes using the ‘enricher’ function from the ‘clusterProfiler’ package in R [62]. GO terms with a *q*-value < 0.1 were considered significantly enriched. The enrichment results were summarized by calculating the gene ratio, defined as the number of DEGs associated with a GO term divided by the total number of input genes, enabling the identification of specific functional categories influenced by gene expression changes while ensuring robustness against multiple hypothesis testing.

For differentially expressed genes (DEGs), annotation was standardized by referencing gene name (if available), locus tag, and annotated function. For DEGs lacking official gene names in both GenBank and RefSeq, putative names or functional descriptions were manually assigned based on homology, including 100% sequence identity using BLASTP searches against the NCBI nonredundant protein database [63] and structure-informed annotations using AlphaFold3 and Foldseek [47, 48]. For instance, MEXAM1_RS04005, originally annotated only as a formyltransferase family protein, showed 100% identity with sequences MCP1543177.1, MCP1561485.1, and MCP1589478.1, and was thus designated as *fmt*.

#### Metabolic pathway analysis and gene essentiality prediction

The GEM of *M. extorquens* AM1, *i*RP911, containing a total of 1142 reactions, was used to correlate GO-enriched DEGs with metabolic functions [19]. On the basis of this model, diagrams of the central carbon metabolism and methionine biosynthesis pathways were constructed. As *i*RP911 was developed prior to the BiGG database, metabolite names were referenced from the *M. extorquens* PA1 model [64, 65]. Abbreviations used in the metabolic pathways are listed in Table S2.

Gene‒protein-reaction (GPR) rules, annotated with the logical operators AND and OR, were used to identify metabolic pathways influenced by DEGs [66]. For DEGs lacking corresponding reactions in the GEM, additional metabolic information was retrieved from KEGG pathway analysis using the ‘KEGGREST’ package in R [67].

Knock-out simulations for *metY* and *kefB* were conducted using cobrapy v.0.29, which is based on the *i*RP911 model, to predict gene essentiality [19, 68]. To specifically simulate the growth of *M. extorquens* AM1 using methanol as the sole carbon source, the GEM was constrained following the procedure from the original reconstruction study [19]. In this condition-specific model, 422 reactions (46 exchange reactions and 376 metabolic reactions) unrelated to the methylotrophic network were inactivated by setting their flux bounds to (0, 0). The objective function was set to maximize the biomass synthesis reaction (reaction ID = ‘BiomassSynthesis’). The lower bound for the methanol exchange reaction (EX_0001) was set to 15.9 ± 1.2 C-mmol·g^−1^·h^−1^, a value based on the mean experimentally measured methanol uptake rate reported for growth at 0.5% methanol [69]. To ensure that other nutrients were not growth-limiting, the flux bounds for all other necessary exchange reactions were set to be unconstrained (−1000 to 1000 mmol·g^−1^·h^−1^) (Table S3). Gene knock-out effects were predicted using the ‘single_gene_deletion’ function, and a gene was defined as essential if the simulation predicted a growth rate of zero upon its deletion.

## Results and discussion

### Minimal medium optimization for *M. extorquens* AM1 prior to ALE

Before starting ALE, the composition of the minimal medium was optimized to enhance the growth of *M. extorquens* AM1 under methanol conditions. First, the medium was supplemented with disodium tungstate. This was done to ensure the maximal activity of formate dehydrogenase 1 (MeFDH1), which, unlike the molybdenum-dependent MeFDH2, specifically requires a tungsten cofactor [42]. While the tungstate concentration has no significant effect on overall cell growth, it is essential for full MeFDH1 activity [42]. Previous studies have demonstrated that MeFDH1 activity requires at least 30 µM disodium tungstate [29, 70]. Therefore, 30 µM disodium tungstate was selected for the modified minimal medium used in all subsequent experiments.

**Fig. 1:**
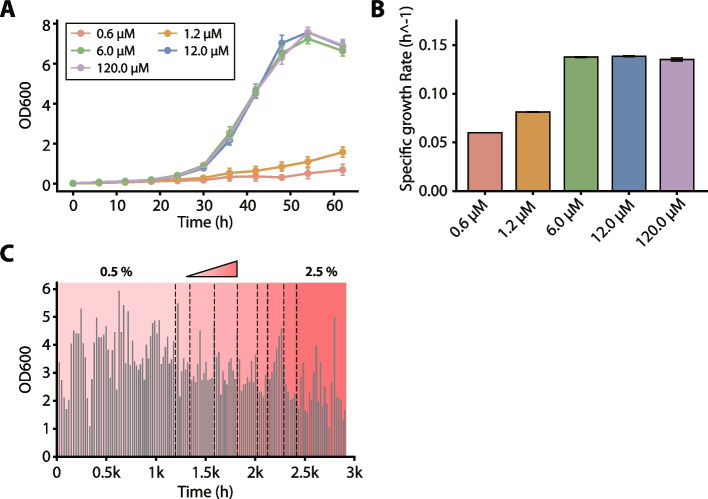
Adaptive laboratory evolution (ALE) of *M. extorquens* AM1 for methanol tolerance, preceded by media optimization. (**A**) Growth curves and (**B**) specific growth rates of the wild-type strain under varying cobalt concentrations (0.6, 1.2, 6.0, 12.0, and 120.0 µM). (**C**) ALE of *M. extorquens* AM1 for methanol tolerance, showing OD_600_ at the point of transfer over successive cultures. The methanol concentration was incrementally increased from 0.5% (v/v; 123.59 mM) to 2.5% (v/v; 617.93 mM), as indicated by the progressively darker background shading. For panels (**A**) and (**B**), data points and error bars represent the mean ± standard deviation from two independent biological replicates (*n* = 2)

Second, growth phenotype assays were performed in this tungstate-supplemented medium to determine the optimal concentration of cobalt, a critical trace element required for methanol metabolism [43, 71]. Elevating the cobalt concentration beyond the standard concentration of 1.2 µM enhanced the specific growth rates by 1.66–1.68-fold and led to a 4.59–4.79-fold improvement in the maximum OD_600_ (Fig. 1A, B). Growth was substantially improved at concentrations exceeding 6.0 µM, with only marginal gains observed at higher levels. Nevertheless, 12.0 µM was selected to ensure that cobalt would not become a limiting factor under methanol conditions. This concentration is consistent with the cobalt level commonly employed in EMC pathway-related methanol metabolism studies [72]. This elevated cobalt concentration likely facilitated the increased biosynthesis of adenosylcobalamin and methylcobalamin. These are essential cofactors required for ethylmalonyl-CoA mutase and methylmalonyl-CoA mutase, which play critical roles in glyoxylate regeneration through the EMC pathway [43, 71, 73].

### Enhancing methanol tolerance in *M. extorquens* AM1 through ALE

ALE of *M. extorquens* AM1 for improved methanol tolerance was carried out by progressively increasing the methanol concentration. The modified minimal medium described above was consistently used throughout the ALE process. The serial Transfer culture started at 0.5% (v/v; 123.59 mM) methanol and gradually increased to 2.5% (v/v; 617.93 mM), leading to the isolation of five evolved strains (Am01-Am05) (Fig. 1C).

**Fig. 2:**
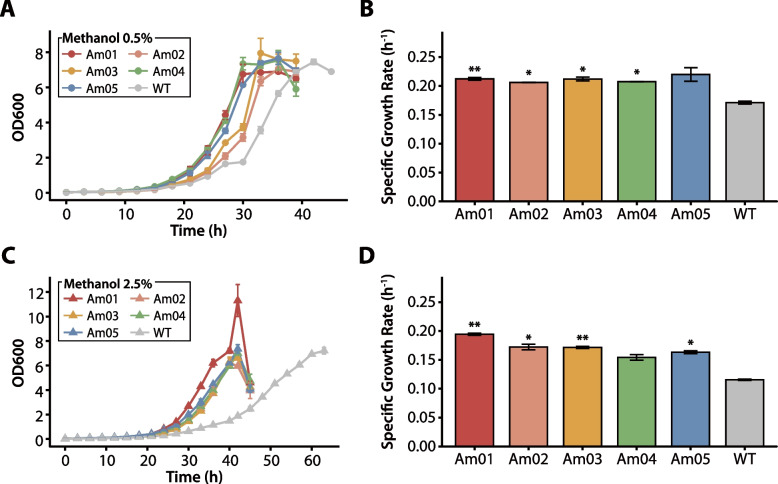
Growth phenotypes of the wild-type and the evolved *M. extorquens* AM1 strains under varying methanol concentrations. (**A**, **C**) Growth curves for the wild-type strain and five evolved strains (Am01-Am05) grown at 0.5% (A, circles) and 2.5% (C, triangles) methanol. The data points represent the mean OD_600_ values ± standard deviations from two independent biological replicates (*n* = 2). (**B**, **D**) Specific growth rates of the wild-type and the evolved strains, measured at 0.5% (**B**) and 2.5% (**D**) methanol concentrations. The error bars represent the standard deviations from two independent biological replicates (*n* = 2). Asterisks denote statistically significant differences compared to the WT strain, as determined by a two-tailed Student's t-test (* *p*-value < 0.05; ** *p*-value < 0.01)

To evaluate the phenotypic characteristics of the evolved strains, growth performance was assessed in both 0.5% and 2.5% methanol to determine whether the adaptations provided benefits across varying methanol concentrations (Fig. 2 and Table 2). In 0.5% methanol, the evolved strains demonstrated a clear growth advantage, with four of the five strains showing statistically significant improvements of 1.21–1.24-fold over the wild-type. The benefits of this adaptation were more pronounced under the stringent 2.5% methanol condition. Am01, Am02, Am03 and Am05 maintained a significant growth advantage over the wild-type. Notably, Am01 exhibited the most substantial improvement in specific growth rate and was highly significant (*p*-value < 0.01). Am03 also demonstrated a highly significant enhancement (*p*-value < 0.01), followed by strains Am02 and Am05, which showed significant improvements (*p*-value < 0.05). The only exception was Am04, which did not show a statistically significant difference from the wild-type in this high-stress environment.

**Table 2:** Growth performance of the evolved strains and engineered strains

Condition	Time interval	Strain	Exponential phase (h)	Specific growth rates (µ, h^-1^)	Fold change vs. WT	Δµ vs. WT	Expected additive Δµ	ε (Epistasis)	Epistasis type	Doubling time (h)	R	Lag time (h)
Methanol 0.5%	3h	Am01	9.00-28.50	0.2124 ± 0.0034	1.24					3.27 ± 0.05	0.9986, 0.9990	2.79 ± 0.36
		Am02	12.00-27.00	0.2061 ± 0.0004	1.21					3.37 ± 0.01	0.9984, 0.9985	4.25 ± 0.18
		Am03	7.50-24.00	0.2121 ± 0.0047	1.24					3.27 ± 0.07	0.9972, 0.9989	3.38 ± 0.28
		Am04	10.50-27.00	0.2073 ± 0.0004	1.21					3.35 ± 0.01	0.9973, 0.9991	1.92 ± 0.04
		Am05	12.00-27.00	**0.2199 ± 0.0165**	1.29					3.17 ± 0.23	0.9988, 0.9995	2.41 ± 0.93
		Wild-type	10.50-27.00	0.1710 ± 0.0034						4.05 ± 0.08	0.9981, 0.9981	0.75 ± 0.35
	4h	mT1	20.00-36.00	0.1803 ± 0.0021	1.13	0.0201				3.85 ± 0.05	0.9994, 0.9996	8.12 ± 0.15
		mT2	12.00-32.00	0.1737 ± 0.0013	1.08	0.0135				3.99 ± 0.03	0.9989, 0.9995	1.47 ± 0.06
		kKO	12.00-32.00	0.1863 ± 0.0030	1.16	0.0261				3.73 ± 0.06	0.9984, 0.9995	2.90 ± 0.09
		kKO + mT1	16.00-36.00	0.1785 ± 0.0038	1.11	0.0183			negative	3.88 ± 0.08	0.9991, 0.9992	8.31 ± 0.04
		kKO + mT2	12.00-32.00	**0.1905 ± 0.0013**	1.19	0.0303			negative	3.65 ± 0.02	0.9992, 0.9993	2.32 ± 0.16
		Wild-type	12.00-32.00	0.1602 ± 0.0042						4.33 ± 0.11	0.9994, 0.9995	0.82 ± 0.47
methanol 2.5%	3h	Am01	21.00-33.00	**0.1944 ± 0.0025**	1.68					3.56 ± 0.04	0.9978, 0.9984	5.61 ± 0.16
		Am02	21.00-37.50	0.1722 ± 0.0068	1.49					4.03 ± 0.16	0.9979, 0.9991	5.30 ± 0.22
		Am03	21.00-39.00	0.1716 ± 0.0025	1.49					4.04 ± 0.06	0.9993, 0.9996	6.21 ± 0.76
		Am04	21.00-39.00	0.1542 ± 0.0068	1.34					4.49 ± 0.20	0.9976, 0.9990	1.91 ± 1.20
		Am05	21.00-34.50	0.1632 ± 0.0034	1.41					4.25 ± 0.08	0.9966, 0.9989	3.18 ± 0.10
		Wild-type	21.00-36.00	0.1155 ± 0.0013						6.01 ± 0.06	0.9979, 0.9983	0.63 ± 0.23
	4h	mT1	20.00-40.00	0.1299 ± 0.0013	1.18	0.0198				5.33 ± 0.06	0.9966, 1.0000	4.60 ± 0.10
		mT2	16.00-36.00	0.1305 ± 0.0132	1.19	0.0204				5.34 ± 0.53	0.9986, 0.9996	1.89 ± 0.91
		kKO	8.00-34.00	0.1206 ± 0.0017	1.10	0.0105				5.75 ± 0.08	0.9982, 0.9995	1.04 ± 0.47
		kKO + mT1	20.00-40.00	0.1377 ± 0.0004	1.25	0.0276	0.0303	−0.0027 ± 0.0042	negative	5.04 ± 0.02	0.9987, 0.9993	5.99 ± 0.02
		kKO + mT2	16.00-38.00	**0.1503 ± 0.0081**	1.37	0.0402	0.0309	0.0093 ± 0.0116	positive	4.62 ± 0.25	0.9995, 0.9999	3.38 ± 1.03
		Wild-type	14.00-32.00	0.1101 ± 0.0055						6.29 ± 0.30	0.9987, 0.9996	0.63 ± 0.83

'Condition' column indicates the methanol concentration used during the experiment

'Time interval' column denotes the interval over which the data were measured

'Strain' column specifies the strain identifier

'Exponential phase (h)' column represents the period during which exponential growth occurred

'Specific growth rates (µ, h⁻^1^)' column indicates the exponential growth rate of each strain

'Fold change vs. WT' column' column shows the ratio of the growth rate compared to the wild-type strain under the same condition

'Δμ vs. WT' column shows the difference in growth rate compared to the wild-type strain under the same condition

'Expected additive Δμ' column represents the theoretical combined effect of two individual mutations, calculated by summing their respective Δμ values

'ε (Epistasis)' column quantifies the interaction effect between mutations by comparing the observed Δμ of the double mutant with the expected additive value (ε = observed − expected)

'Epistasis type' column classifies the nature of interaction as positive (synergistic) or negative (antagonistic), based on the sign of ε

'Doubling time (h)' column refers to the time required for the population to double

'R' column indicates the correlation coefficient of growth rate estimation

'Lag time (h)' column denotes the duration before exponential growth initiation

^a^All values represent the mean ± standard deviation from two biological replicates (*n* = 2)

^b^Boldface indicates the highest specific growth rate within each experimental group

### Identification and characterization of key adaptive mutations conferring methanol tolerance

#### Genome resequencing and mutation analysis

**Fig. 3:**
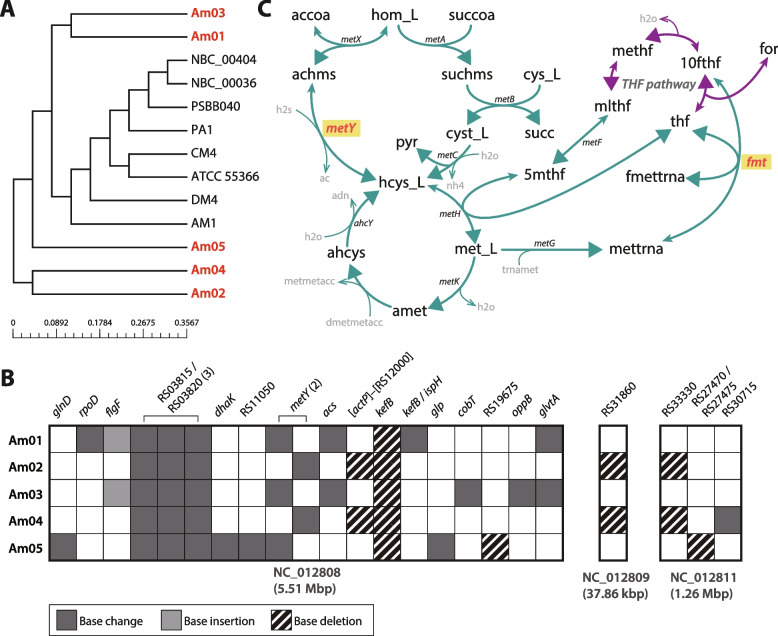
Comparative genomic analysis of the evolved strains. (**A**) Phylogenetic analysis of the five evolved strains (Am01-Am05, highlighted in red) and eight additional *M. extorquens* strains with publicly available coding sequences (CDSs). The phylogenetic tree was constructed using the neighbor‒joining method. (**B**) Heatmap illustrating the distribution of mutations across the genomes of the five evolved strains. Mutations are categorized as base changes, base insertions, and base deletions, each color-coded for clarity. Gene locus tags are abbreviated as unique identifiers (excluding the ‘MEXAM1_’ prefix), and for intergenic mutations, loci of adjacent genes are separated by a slash. The numbers in parentheses indicate multiple mutations of the same type within a single gene. (**C**) Interconnected methionine biosynthesis and the THF pathway in *M. extorquens* AM1. The diagram illustrates the conversion of *O*-acetyl-L-homoserine (achms) to methionine (met_L) through intermediates such as L-homocysteine (hcys_L), with contributions from the tetrahydrofolate (THF) pathway (purple), which integrates C1 units into methionine biosynthesis. Metabolites are labeled for clarity, with cofactors labeled in small gray font. Genes are labeled in smaller black font, and DEGs are highlighted in yellow boxes. The *metY* gene, which catalyzes the conversion of *O*-acetyl-L-homoserine to L-homocysteine using H₂S, is highlighted because of its mutation in evolved strains. Additionally, the putative *fmt* gene (MEXAM1_RS04005), identified as a differentially expressed gene, is included in the pathway to represent its role in methionine biosynthesis. Larger solid arrows represent the forward direction of enzymatic reactions, whereas smaller solid arrows indicate reverse reactions. The open arrows indicate the transfer or involvement of cofactors and small molecules. Abbreviations from the BiGG database are summarized in Table S2

To explore the genetic basis of these adaptive traits, pan-genome analysis was conducted by comparing the genomes of the five evolved strains with those of eight reference *M. extorquens* strains. This analysis revealed an open pan-genome, indicating high genetic diversity and the potential for novel gene variants to arise under evolutionary pressure (Fig. S2). A total of 43,447 genes were identified and categorized into 3,792 gene clusters in the core genome (70.17%), 1,834 gene clusters in the accessory genome (16.45%), and 5,677 unique gene clusters (13.39%). The high genomic similarity to wild-type indicates that methanol tolerance resulted from minor genetic changes rather than extensive genomic rearrangements (Fig. 3A).

In addition, a total of 23 mutations were identified across the evolved strains (Fig. 3B). These included 16 single nucleotide polymorphisms (SNPs), 5 small indels, and 2 large deletions. These mutations affected 13 individual core genes, with 1 large deletion spanning two additional core genes. Two mutations occurred in accessory genes, and five mutations occurred in intergenic regions. The number and types of mutations varied among strains, with Am01 and Am03 having the most mutations and Am02 having the fewest. To identify positively selected mutations potentially contributing to methanol tolerance, it was focused on mutations observed across all evolved strains. Notably, mutations were commonly found in *metY* (MEXAM1_RS11880), and identical mutations were detected in *kefB* (MEXAM1_RS12770) and in the intergenic region between MEXAM1_RS03815 (16S rRNA) and MEXAM1_RS03820 (M23 family metallopeptidase).

**Fig. 4:**
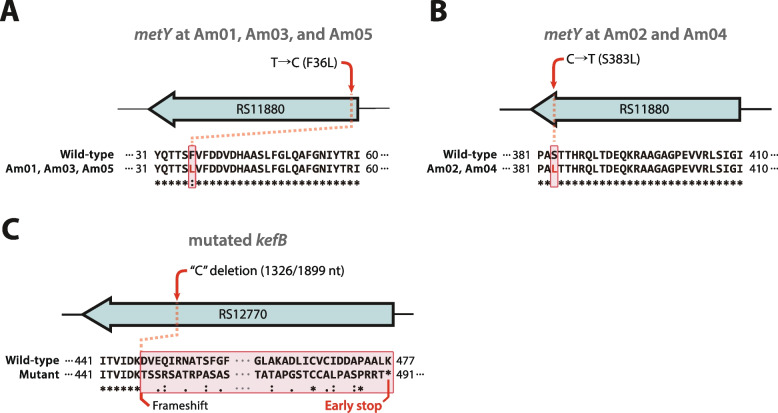
Schematic diagrams illustrating specific mutations in key genes of the evolved strains. (**A**) A base change mutation in *metY* in the Am01, Am03, and Am05 strains resulted in the amino acid substitution F36L. (**B**) A base change mutation in *metY* in the Am02 and Am04 strains resulted in the amino acid substitution S383L. (**C**) A base deletion mutation in *kefB* led to a frameshift mutation. The mutation sites are indicated with red arrows, and affected protein regions are connected by red dotted lines. The corresponding altered amino acid residues are highlighted with red boxes

Two distinct amino acid substitutions were identified in *metY*: F36L (Am01, Am03 and Am05) and S383L (Am02 and Am04) (Fig. 4A, 4B). The *metY* gene encodes *O*-acetyl-L-homoserine sulfhydrylase, an essential enzyme in methionine biosynthesis (Fig. 3C) [74]. This enzyme functions as a homotetramer in its active state, which is required for its catalytic activity [51, 75]. Although the asymmetric unit typically consists of a homodimer, the functional enzyme is formed by the symmetric association of two such dimers. Each monomer binds a pyridoxal 5ʹ-phosphate (PLP) cofactor, which is covalently linked to the catalytic lysine residue and essential for enzyme function [76].

A 1 bp deletion at position 1,326 in *kefB* led to a frameshift mutation, introducing a premature stop codon at residue 478 (Fig. 4C). The *kefB* gene encodes a transmembrane potassium efflux antiporter that plays a role in regulating intracellular pH and ionic homeostasis [77, 78]. Domain analysis indicated that the cation/H^+^ exchanger domain (IPR006153, residues 18–382) remained intact, whereas the N-terminal portion of the regulator of K^+^ conductance (IPR003148, residues 413–535) was truncated. This domain is known to mediate the stress-responsive gating of potassium efflux, particularly under acidic conditions. Although a portion of the domain remains, truncation of its N-terminal segment likely compromises domain folding or disrupts key regulatory interactions, thereby impairing pH-dependent gating. Given the structural disruption within the regulatory module, the mutation is expected to reduce or abolish *kefB*-mediated potassium efflux under acidic stress conditions caused by methanol metabolism.

In addition to the mutations in coding regions, a recurrent mutation in the intergenic region between the 16S rRNA gene (MEXAM1_RS03815) and an M23 family metallopeptidase (MEXAM1_RS03820) was identified in all five evolved strains, highlighting its potential adaptive importance. The location of this mutation suggests that it might affect the expression of the adjacent metallopeptidase gene. However, a subsequent analysis of gene expression revealed no significant transcriptional changes for MEXAM1_RS03820 in the evolved strains. Therefore, the adaptive benefit of this mutation may stem from other mechanisms, such as effects on post-transcriptional regulation or local DNA topology. The precise role of this recurrent intergenic mutation remains an interesting topic for future investigation.

#### Structural prediction and residue-level analysis of MetY mutations

To gain structural insight, structural modeling using AlphaFold3 was employed (Additional file 1) [47]. The protein was modeled in its known homotetrameric state, with the four subunits distinguished by color for clarity (Fig. S3A). The reliability of the resulting model was very high, with an average per-residue confidence score (pLDDT) of 96.24. The visualization of the pLDDT score revealed consistently high confidence throughout the structure, with only slight, localized decreases in flexible surface loops (Fig. S3B). Analysis of this high-confidence model revealed that the two substitutions occurred at distinct positions within the structure [47]. Residue F36 is located within a flexible loop near the hydrophobic core, close to potential monomer‒monomer interaction sites, whereas residue S383 resides in a surface-exposed α-helix. These structural differences may influence enzyme activity or structural stability under elevated methanol concentrations.

To identify experimentally resolved reference protein structures, Foldseek-based structural alignment was conducted. Among the identified homologs, the *Thermotoga maritima* MetY structure (PDB: 7KB1) was selected for comparison, as it forms a homotetramer and includes a bound PLP cofactor in chain D [51]. The calculated TM-score between *M. extorquens* MetY and *T. maritima* MetY was 0.8880, indicating a high degree of structural similarity (Fig. S3C). To examine potential interactions between the mutated residues and the PLP cofactor, the distances from F36 and S383 to the PLP molecule within the asymmetric unit of *M. extorquens* MetY were measured (Fig. S3C). The closest distance was 14.29 Å, suggesting that neither residue directly interacts with PLP or contributes to its stabilization [79, 80]. Although F36L and S383L are spatially distant from the PLP-binding or catalytic sites, these mutations were consistently observed in the evolved strains.

To complement structural modeling, the physicochemical properties of the mutated residues and their evolutionary conservation were analyzed to assess their potential functional impact. The F36L mutation replaces phenylalanine (aromatic, bulky, and hydrophobic) with leucine (nonaromatic, aliphatic, and hydrophobic), which may affect local packing interactions within the hydrophobic core. In contrast, the S383L mutation replaces serine (polar, small) with leucine (hydrophobic, larger side chain), which introduces a pronounced shift in both the polarity and side chain. Given that S383 is located in a surface-exposed α-helix, this change may disrupt methanol interactions or local secondary structure stability.

These residue-level changes, combined with their recurrence across evolved strains, suggest a possible role in fine-tuning enzyme stability or suppressing side reactions under methanol stress. Although the two substitutions are structurally distant, both converged on leucine and consistently occurred, suggesting possible similar adaptive roles in fine-tuning enzymatic function under methanol stress. This convergence may reflect the physicochemical tolerance of leucine, a small, hydrophobic, and helix-compatible residue. While such substitutions could provide subtle structural stabilization, the recurrence of leucine at distinct sites may also be coincidental. The independent emergence of *metY* and *kefB* mutations across all five evolved strains highlights their potential roles as core adaptive targets under methanol selection pressure. In particular, the recurrence of two distinct substitutions in *metY* (F36L and S383L) and an identical frameshift truncation in *kefB* suggest convergent evolutionary solutions to methanol-induced stress.

#### Phenotypic characterization of engineered strains

To assess the functional roles of the recurrent mutations, a predictive approach using the GEM, *i*RP911 were first employed [19]. While GEMs can simulate responses to varying methanol concentrations, standard flux balance analysis, by its nature, does not account for substrate inhibition kinetics that can occur in vivo. This limitation was evident in preliminary simulations where increasing the methanol concentration resulted only in a proportional scaling of metabolic fluxes, failing to capture known growth bottlenecks. Therefore, to ensure a physiologically relevant basis for analysis, a single, experimentally measured methanol uptake rate under methanol 0.5% was used as a fixed constraint for all subsequent gene knock-out simulations. Under this constraint, the simulation predicted that knocking out *metY* would result in no growth, confirming its essentiality for cell viability. This prediction was subsequently validated experimentally, as allelic exchange required methionine supplementation (Table S4 and S5; see Sect. "Genetic modifications and functional validation" for details) [81]. In contrast, knocking out *kefB* was predicted to result in a biomass objective value identical to that of the wild-type strain, suggesting that *kefB* is nonessential under the given conditions.

Given its essential role predicted by GEM analysis, *metY* was not knocked out but was further investigated through targeted SNP modifications. Point mutations F36L and S383L were introduced to generate the engineered strains mT1 and mT2, respectively. To ensure mutant viability during allelic exchange, methionine supplementation was required (Tables S4 and S5), thereby allowing construction of the merodiploid without an associated fitness cost. In the case of *kefB*, the identified 1 bp deletion was predicted to cause a frameshift, leading to a truncated protein and a complete loss of function. Therefore, to directly test the phenotypic effect of inactivating the KefB protein, a complete knock-out was constructed as a proxy for this frameshift mutation. This strategy enables the evaluation of whether removing potassium efflux improves methanol tolerance by relieving the cellular energy burden. Furthermore, two double mutant strains, kKO + mT1 and kKO + mT2, were constructed to assess the potential combined effects of these mutations.

**Fig. 5:**
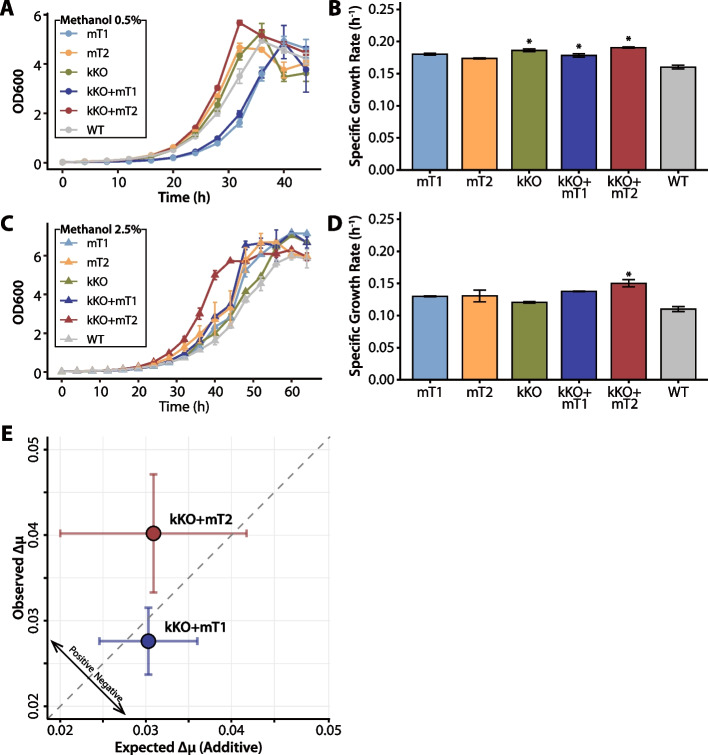
Growth phenotypes and genetic interactions of engineered *M. extorquens* AM1 strains under varying methanol concentrations. (**A**, **C**) Growth curves of the wild-type *M. extorquens* AM1 and five engineered strains: two with mutations in *metY* (mT1, mT2), one with *kefB* knock-out (kKO), and two with combined mutations in *metY* and *kefB* (kKO + mT1, kKO + mT2), grown at 0.5% (A, circles) and 2.5% (C, triangles) methanol concentrations. The data points represent the mean OD_600_ values ± standard deviations from two independent biological replicates (*n* = 2). (**B**, **D**) Specific growth rates (µ) at 0.5% (**B**) and 2.5% (**D**) methanol. Bars represent the mean, and error bars represent the standard deviations from two independent biological replicates (*n* = 2). (**E**) Epistasis analysis comparing the observed specific growth rates (µ) against the expected additive effects of single mutations in 2.5% methanol. The dashed line represents the theoretical additive expectation, assuming no interaction between mutations. Data points above this line suggest positive epistasis, whereas those below indicate negative epistasis. Error bars represent the propagated standard error. **Asterisks indicate a statistically significant difference compared to the wild-type strain, determined by a two-tailed Student’s t-tests (*p-value < 0.05)

To validate the functional roles of the identified mutations, the growth phenotypes of the engineered strains were characterized (Fig. 5 and Table 2). At 0.5% methanol, the mutations exhibited distinct effects. The *kefB* knock-out strain (kKO) conferred a statistically significant growth advantage over the wild-type (*p*-value < 0.05), whereas the single *metY* point mutations, mT1 and mT2, did not (Fig. 5B). When combined with the *kefB* knock-out, both double mutants (kKO + mT1 and kKO + mT2) also showed significantly increased growth rates. These three strains (kKO, kKO + mT1, and kKo + mT2) demonstrated 1.11–1.19-fold improvements in specific growth rate compared to the wild-type.

Under the more stringent 2.5% methanol condition, the intensified selective pressure revealed a clearer hierarchy of methanol tolerance. Notably, only the double mutant strain kKO + mT2 maintained a statistically significant growth advantage over the wild-type (*p*-value < 0.05), achieving a 1.37-fold increase in its specific growth rate (Fig. 5D). All other engineered strains, including the single mutants and the kKO + mT1 double mutant, did not show a statistically significant difference from the wild-type under this elevated methanol concentration. This finding highlights the superior combinatorial benefit of the S383L *metY* mutation (mT2) when paired with the *kefB* knock-out for enhancing methanol tolerance.

#### Genetic interaction analysis and mechanistic interpretation

The combinatorial benefit was further investigated through an epistasis analysis, comparing the observed growth rates of double mutants to the expected additive effects of the single mutants. While the double mutants exhibited superior methanol tolerance compared to the single mutants, this enhanced fitness was found to be largely additive rather than synergistic under 2.5% methanol (Fig. 5E and Table 2). Neither the minor positive deviation for kKO + mT2 (ε = 0.0093 ± 0.0116) nor the minor negative deviation for kKO + mT1 (ε = −0.0027 ± 0.0042) was statistically significant. This additivity strongly suggests that the two mutants confer resilience through independent and mechanistically distinct pathways.

A plausible hypothesis for the *metY* mutation is that they enhance methanol tolerance by fine-tuning enzyme activity. It is well-established that in addition to its primary role in methionine biosynthesis, MetY can catalyze a side reaction with excess methanol to produce the toxic analog methoxine, a major contributor to methanol toxicity [13, 82, 83]. Although the growth improvements conferred by the single *metY* mutations did not reach statistical significance in this study, a consistent trend of enhanced fitness compared to the wild-type was observed (Fig. 5B, 5D). This discrepancy between the evolved strains and the engineered single mutants may suggest that the adaptive benefit of the *metY* mutation is fully realized only in combination with other mutations acquired during ALE, or its effect is subtle and becomes more pronounced when coupled with the energy-saving *kefB* knock-out. This observation, combined with the known toxicity of its byproduct, leads to the plausible hypothesis that the F36L and S383L substitutions mitigate methanol toxicity by either reducing the rate of this side reaction or enhancing the overall stability and efficiency of the main reaction of MetY. This contrasts with previous studies where other nonsynonymous mutations in *metY* were associated with general enzyme inactivation, reducing both its main reaction (conversion of *O*-acetyl-L-homoserine to L-homocysteine) and side reaction (conversion to methoxine) [34].

The inactivation of *kefB* represents another key adaptive strategy centered on energy conservation. The observed 1 bp deletion led to a frameshift and premature translation termination, likely abolishing its antiporter function. This loss of function would eliminate the energetic cost of potassium efflux. This process may be rendered less essential under methanol stress, where the accumulation of acidic intermediates could naturally contribute to pH buffering [84]. Consistent with this hypothesis, previous studies have shown that eliminating unnecessary ion transport can redirect cellular energy toward methanol-specific metabolic processes, enhancing overall fitness [85, 86].

Therefore, the integration of these two distinct adaptive mechanisms, one mitigating toxicity and the other conserving energy, demonstrates an effective strategy for achieving enhanced stress resilience, even in the absence of positive epistasis. The observation that two mutations affecting different cellular processes together provided greater benefits suggests that methanol tolerance requires comprehensive cellular responses. To further validate the observed interaction, future experiments could assess intracellular ATP levels and translation activity in single versus double mutants under methanol stress. This would clarify whether energy conservation from potassium efflux modulation directly supports enhanced biosynthetic capacity.

### Transcriptomic overview and functional insight

#### Global transcriptional patterns

**Fig. 6:**
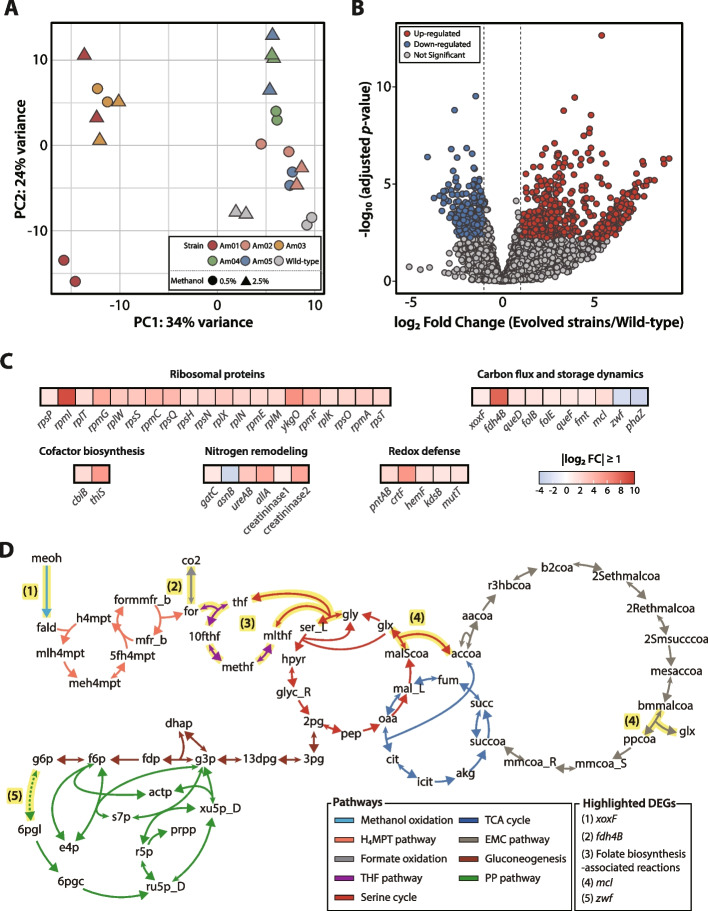
Transcriptomic analysis of the wild-type and the evolved strains under various methanol concentrations. (**A**) PCA of transcriptomic data from wild-type and the evolved strains (Am01–Am05) at 0.5% and 2.5% methanol concentrations. (**B**) Volcano plot showing differentially expressed genes (DEGs) based on the interaction between strain type (evolved strains vs. wild-type) and methanol concentration (0.5% vs. 2.5%). DEGs were identified using DESeq2, with significantly upregulated genes (red dots) and downregulated genes (blue dots) defined by a threshold of absolute value of log_2_ fold change > 1 and adjusted *p*-value < 0.05. Nonsignificant genes, including those excluded due to specific conditions in the analysis, are shown in gray. (**C**) Heatmap of selected DEGs involved in ribosomal protein synthesis and diverse metabolic pathways. Genes with an absolute log_2_ fold change > 1 are shown in red, whereas those with values < 1 are depicted in blue. The gradient reflects the relative intensity of expression changes. (**D**) Central carbon metabolic pathways of *M. extorquens* AM1. Each pathway is color-coded, and reactions influenced by up- or downregulated DEGs are shown as solid or dotted lines, respectively. Reactions directly influenced by DEGs are highlighted with yellow backgrounds. Larger arrows represent the forward reaction directions, whereas smaller arrows indicate reverse reactions. Genes differentially expressed in evolved strains, including *xoxF* (1)*, fdh4B* (2), folate biosynthesis-associated genes (3), *mcl* (4), and *zwf* (5), are indicated. The corresponding reactions are labeled (1)-(5). Abbreviations from the BiGG database are summarized in Table S2

Building on the findings from ALE, a transcriptomic analysis was performed to elucidate the mechanisms underlying enhanced methanol tolerance. A dual-factor interaction approach integrated both methanol concentration and adaptive traits as interacting variables to identify differentially expressed genes (DEGs) influenced by their interplay [57, 87]. PCA revealed distinct transcriptional profiles among the wild-type and the evolved strains, with strain identity and methanol concentration as the primary drivers of variance (Fig. 6A). A total of 767 DEGs were identified, including 475 upregulated and 292 downregulated genes (Fig. 6B).

Additionally, Gene Ontology (GO) enrichment analysis categorized these DEGs into biological processes (BP), cellular components (CC), and molecular functions (MF), highlighting important functional changes associated with methanol tolerance (Fig. S4) [60]. The upregulated DEGs were primarily enriched in translation-related processes, including Translation (GO:0006412, BP), Ribosome (GO:0005840, CC), and Structural constituent of ribosome (GO:0003735, MF). Notably, the upregulation of 20 genes encoding 30S and 50S ribosomal proteins indicated enhanced ribosomal assembly and protein synthesis under elevated methanol concentrations (Fig. 6C and Table S6). This enrichment in translation-related genes may reflect the downstream effect of adaptive *metY* mutations. By relieving methoxine-associated toxicity and restoring methionine availability, the *metY* mutation likely removed a bottleneck in amino acid supply, allowing increased ribosome biogenesis and translation activity under methanol stress. Among these, the shortest 50S ribosomal proteins, L35 (*rpmI*) and L36 (*ykgO*), exhibited the greatest upregulation, with L35 showing the greatest log_2_ fold change of 9.09. The pronounced upregulation of these small ribosomal proteins suggests their critical role in ribosome stability and efficiency during methanol adaptation. L36, a zinc-binding ribosomal protein, likely contributes to structural stabilization through zinc coordination, whereas L35 may play a key role in ribosomal assembly under stress conditions despite lacking a metal-binding domain [88].

In contrast, the downregulated DEGs were predominantly depleted in GO terms related to nucleotide binding, such as ATP binding (GO:0005524, MF), Ribonucleotide binding (GO:0032553, MF), and Purine nucleotide binding (GO:0017076, MF). This downregulation suggests possible changes in nucleotide-related processes under elevated methanol concentrations.

#### Carbon flux and storage dynamics

To further understand the metabolic implications, transcriptomic data were integrated with the KEGG database and the GEM. Among the 767 DEGs, 73 genes were mapped to reactions in the GEM, indicating their functional relevance to core metabolic processes (Table S6). These central carbon metabolic pathways have been previously characterized in detail (Fig. 6D) [89].

Methanol assimilation and detoxification are central to the survival of *M. extorquens* AM1 under elevated methanol concentrations. Several genes directly involved in methanol oxidation were significantly upregulated. The most notable gene was *xoxF* (MEXAM1_RS08325, lanthanide-dependent methanol dehydrogenase, log₂FC = 1.18), which facilitates the oxidation of methanol to formaldehyde [90]. XoxF catalyzes the oxidation of methanol to formaldehyde, and its upregulation under elevated methanol concentrations suggests accelerated metabolic flux through the methanol oxidation pathway, enhancing formaldehyde turnover and reducing potential toxicity. Additionally, *fdh4B* (MEXAM1_RS09885, subunit B of MeFDH4, log₂FC = 8.02) was upregulated, supporting the oxidation of formate to CO₂ [90, 91]. MeFDH4 oxidizes formate to CO₂, preventing cytoplasmic acidification and contributing to redox homeostasis. By efficient conversion of methanol to formaldehyde and subsequently to CO₂, these enzymes likely reduce toxic intermediate accumulation and mitigate oxidative stress, thereby promoting overall redox balance.

The activation of the tetrahydrofolate (THF) pathway was evident in the evolved strains, as indicated by the upregulation of folate biosynthesis genes, including *queD* (MEXAM1_RS04775), *folB* (MEXAM1_RS08345), *folE* (MEXAM1_RS10690), and *queF* (MEXAM1_RS14060) (log₂FC = 1.69, 1.59, 1.30, and 1.34, respectively) [92]. THF derivatives serve as key factors in C1 metabolism, supporting both formaldehyde detoxification and methionine biosynthesis. Their increased biosynthesis suggests an increased demand for THF-dependent methylation reactions, which integrate C1 units into central carbon metabolism. In particular, THF-dependent reactions play a pivotal role in directing carbon flux toward methionine biosynthesis, as they provide 5-methyl-THF, a key methyl donor required for L-homocysteine conversion to L-methionine (Fig. 3C). This metabolic coupling between C1 metabolism and methionine biosynthesis highlights the integrative nature of the evolved adaptive response [92].

Moreover, putative *fmt* (MEXAM1_RS04005, methionyl-tRNA formyltransferase, log₂FC = 1.23) was also upregulated, playing a critical role in the incorporation of formyl-methionine into nascent polypeptides during translation initiation [93]. This enzyme is necessary for bacterial translation initiation and further links folate metabolism with protein biosynthesis, emphasizing the necessity of methionine availability for the synthesis of both ribosomal and stress-responsive proteins during methanol adaptation. Additionally, *mcl* (MEXAM1_RS08290, malyl-CoA lyase and beta-methylmalyl-CoA lyase, log₂FC = 1.91) was upregulated [94]. These enzymes play a vital role in glyoxylate assimilation within the serine cycle and EMC pathway, facilitating carbon flux regulation and optimizing C1 metabolism.

In addition to its role in formaldehyde detoxification and C1 incorporation, redox balance plays a critical role in methanol adaptation. The upregulation of *xoxF* and *fdh4B* indicates a reinforced methanol–formaldehyde–formate oxidation process. This metabolic shift likely increased intracellular NADH levels, as formate oxidation to CO₂ is NAD⁺-dependent. The resulting elevated NADH pool likely triggered broader adaptive responses to maintain intracellular redox homeostasis. Within central carbon metabolism, a prominent adaptation was the downregulation of *zwf* (MEXAM1_RS11790, glucose-6-phosphate dehydrogenase, log₂FC = −2.43), the major enzyme generating NADPH in the oxidative phase of the pentose phosphate (PP) pathway. Such repression may reflect reduced cellular requirements for NADPH under conditions favoring NADH-centric metabolism.

Carbon storage dynamics may also play a role in methanol adaptation. Notably, *phaZ* (MEXAM1_RS21730, polyhydroxyalkanoate depolymerase, log₂FC = −3.08), involved in poly-β-hydroxybutyrate (PHB) metabolism, was significantly downregulated [95]. This finding suggests a potential shift toward reduced PHB degradation. Although PHB levels were not directly quantified in this study, PHB is known to function as a carbon and redox buffer that enhances bacteria survival under stress conditions [96, 97].

#### Cofactor-dependent enzyme regulation

In addition to these primary metabolic pathways, several DEGs that indirectly contribute to C1 metabolism through cofactor biosynthesis and enzyme regulation were identified. In EMC pathway, the upregulation of *cbiB* (MEXAM1_RS24520, adenosylcobinamide-phosphate synthase, log₂FC = 2.00) underscores the importance of cobalamin-dependent reactions as an adaptive strategy to increase carbon recycling efficiency [72]. This upregulation appears to be connected to the optimized cobalt levels in the medium (as described in Sect. "Minimal medium optimization for *M. extorquens* AM1 prior to ALE"), which may have facilitated the increased biosynthesis of adenosylcobalamin and methylcobalamin, essential cofactors required for ethylmalonyl-CoA mutase and methylmalonyl-CoA mutase. These enzymes play critical roles in glyoxylate regeneration through the EMC pathway and efficient carbon flux redistribution, thereby maintaining metabolic balance and likely supporting methionine biosynthesis under methanol stress (Fig. 6D) [73].

Alongside cofactor support for carbon recycling, adaptations in sulfur metabolism also emerged as a key strategy to support methionine biosynthesis and cellular stability under methanol stress. The expression of *thiS* (MEXAM1_RS02060, sulfur carrier protein, log₂FC = 5.55) was strongly upregulated. This gene is involved in thiamine biosynthesis, which is essential for supplying thiamine pyrophosphate (TPP), a cofactor required for multiple key metabolic enzymes, including those involved in glycolysis and the TCA cycle [98]. Moreover, since *metY* catalyzes L-homocysteine formation from *O*-acetyl-L-homoserine through a sulfhydrylation step, the increased sulfur assimilation by *thiS* could further support methionine biosynthesis, indirectly bridging cofactor-level changes with the *metY*-driven pathway for methionine under methanol stress. The substantial upregulation of *thiS* is particularly noteworthy, as it contributes to both sulfur metabolism and TPP biosynthesis, suggesting an important role in mitigating methanol toxicity through enhanced metabolic enzyme function.

#### Stress mitigation through nitrogen remodeling and redox defense

The transcriptomic analysis also revealed notable adaptive changes in nitrogen metabolism. The upregulation of *gatC* (MEXAM1_RS16730, subunit C of glutamyl-tRNA and aspartyl-tRNA amidotransferases, log₂FC = 1.31) indicates a shift toward increased utilization of glutamate and aspartate for tRNA charging [99–101]. Conversely, the downregulation of putative *asnB* (MEXAM1_RS25255, asparagine synthase, log₂FC = −2.88) suggests a reduced demand for asparagine biosynthesis. This potentially leads to the reallocation of glutamine to other nitrogen-dependent processes, such as glutamate synthesis and essential amino acid production, thereby contributing to methanol stress adaptation [102, 103]. The upregulation of *ureAB* (MEXAM1_RS04630, bifunctional urease subunit gamma/beta, log₂FC = 3.07), *allA* (MEXAM1_RS07980, ureidoglycolate lyase ammonia, log₂FC = 4.71), and creatininase genes (creatininase1 = MEXAM1_RS10035 and creatininase2 = MEXAM1_RS19285, log₂FC = 1.04 and 4.89, respectively), which are involved in creatinine degradation, further supports alternative nitrogen recycling, facilitating adaptive nitrogen assimilation pathways [104–106]. This buffering mechanism not only regulates pH but also facilitates the integration of glyoxylate, a key intermediate produced during nitrogen recycling, into central metabolic pathways such as the serine cycle and the EMC pathway.

In addition to nitrogen metabolism, several adaptive strategies beyond central carbon metabolism were identified to maintain redox balance under methanol stress. The elevated intracellular NADH levels, resulting from enhanced formate oxidation with *fdh4B,* appear to have driven further adaptive responses. The increased expression of *pntAB* (MEXAM1_RS13930, proton-translocating NAD(P) transhydrogenase, log₂FC = 2.09), which is known as the major source of NADPH, likely facilitates the conversion of excess NADH into NADPH, thereby stabilizing redox cofactors and alleviating potential redox imbalances [107]. This redistribution of reducing equivalents allowed for a reduced reliance on endogenous NADPH production through the oxidative phase of the PP pathway, which was consistent with the observed downregulation of *zwf* (as described in Sect. "Carbon flux and storage dynamics"). This strategy of utilizing the proton-translocating transhydrogenase PntAB to convert excess NADH into NADPH appears to be a key mechanism for maintaining redox homeostasis under metabolic stress. Notably, a similar reliance on transhydrogenases for redox balancing has been studied in other metabolically robust bacteria, such as *Pseudomonas putida*, when facing oxidative stress during the biodegradation of aromatic compounds [38]. However, unlike *P. putida* which simultaneously upregulates the PP pathway, *M. extorquens* appears to favor a more direct conversion of NADH to NADPH through PntAB while conserving resources by repressing *zwf*. The emergence of this refined strategy in the evolved strains highlights an efficient and convergent evolutionary solution to manage the high NADH flux inherent to methylotrophy.

Moreover, the upregulation of genes related to oxidative stress mitigation, such as *crtF* (MEXAM1_RS13770, carotenoid methyltransferase, log₂FC = 5.66) and *hemF* (MEXAM1_RS02400, oxygen-dependent coproporphyrinogen III oxidase, log₂FC = 1.90), suggests that comprehensive cellular strategies to mitigate reactive oxygen species (ROS) stress are likely exacerbated by increased respiratory activity and excess NADH [108, 109]. Carotenoid biosynthesis mitigates ROS-induced oxidative damage, whereas heme is essential for the cytochrome and catalase enzymes involved in ROS detoxification and cellular respiration.

In addition to these strategies for mitigating internal oxidative stress, the evolved strains also exhibited adaptations to reinforce cellular integrity. The upregulation of *kdsB* (MEXAM1_RS01105, 3-deoxy-D-manno-octulosonate cytidylyltransferase, log₂FC = 1.41), which is involved in lipopolysaccharide biosynthesis, implies structural reinforcement of the cell envelope. This adjustment potentially mitigates membrane damage induced by elevated methanol concentrations, ensuring continued metabolic and respiratory activity under stress conditions [110]. Similarly, putative *mutT* (MEXAM1_RS00950, (deoxy)nucleoside triphosphate pyrophosphohydrolase) was upregulated (log₂FC = 1.65), indicating its role in degrading oxidized nucleotides and maintaining DNA integrity under stress. The coordinated upregulation of these genes demonstrates a comprehensive strategy to counter ROS-induced damage, which is a major component of methanol toxicity [111, 112].

Transcriptomic remodeling is likely not independent of genetic adaptation and may reflect downstream effects of specific core mutations, although direct causal relationships remain to be experimentally established. For example, *metY* mutations coincided with the upregulation of putative *fmt* and *thiS*, supporting sulfur assimilation and enhancing translation initiation components. This was accompanied by the upregulation of 20 ribosomal protein genes, suggesting enhanced translational capacity under methanol stress. In parallel, the frameshift mutation in *kefB* likely reduced potassium efflux. This conserved energy may have been reallocated to support other adaptive transcriptional responses, including ROS detoxification and metabolic reconfiguration.

### Limitations and future directions

While this study provides comprehensive systems-level insights into methanol adaptation, several limitations and directions for future work should be noted. The proposed effects of *metY* mutations, on the basis of their biochemical function, mutational context, and phenotypic consequences, remain hypothetical. Future studies should experimentally validate these predictions with enzyme kinetic assays on mutant MetY, focusing on substrate specificity toward methoxine. In addition, high-resolution structural methods such as X-ray crystallography would help confirm the predicted structural changes induced by F36L and S383L. Although *kefB* disruption improved fitness, its precise role in modulating bioenergetics under methanol stress remains unclear. Direct measurements of the membrane potential or intracellular ATP levels will be necessary to clarify its mechanistic contribution. Such studies will be essential for applying these findings into targeted strategies for industrial strain optimization.

Furthermore, the functional significance of the recurrent mutation in the intergenic region between the 16S rRNA and a metallopeptidase gene remains unresolved. Its consistent emergence across all evolved strains strongly suggests an adaptive role that was not captured by transcriptomic analysis of the adjacent gene. Future studies could investigate its impact on local DNA topology, non-coding RNA expression, or post-transcriptional regulation to uncover this novel adaptive mechanism. This interconnected and systems-level understanding provides a comprehensive foundation for rational engineering strategies that target multiple cellular processes simultaneously to achieve robust methanol tolerance.

## Conclusion

This study demonstrates that enhanced methanol tolerance in *M. extorquens* AM1 is not the result of a single adaptation but emerges from the convergence of multiple, mechanistically distinct strategies. Pivotal mutations in *metY* were hypothesized to mitigate toxicity by fine-tuning methionine biosynthesis, while mutations in *kefB* likely conserve cellular energy by reducing potassium efflux. This genetic framework was supported by a coordinated transcriptional remodeling that optimized C1 metabolism, redox balance, and translational capacity. The additive integration of these independent strategies highlights a robust evolutionary solution to methanol stress, providing a systems-level foundation for rational engineering.

The evolved strains demonstrate significant potential for sustainable biomanufacturing. Specifically, their enhanced tolerance allows for higher methanol loading in bioreactors, which can increase volumetric productivity and reduce the operational costs associated with cooling and substrate feeding, thereby improving the economic viability of methanol-based bioprocesses. The identified mutations in *metY* and *kefB* may be particularly useful targets for strain engineering aimed at simultaneously optimizing metabolic throughput and stress resistance, two key bottlenecks in methanol-based fermentation systems.

While challenges remain in maintaining strain stability under dynamic industrial conditions, future research should focus on functionally validating the identified mutations. In particular, biochemical characterization of mutant MetY enzymes (e.g., substrate specificity and methoxine formation) and biophysical assays assessing membrane potential and intracellular ATP levels in *kefB*-disrupted strains will clarify their mechanistic contributions. Testing these engineered strains in continuous and fed-batch systems will also be essential for evaluating their long-term performance and robustness under industrially relevant conditions. Furthermore, the multilayered systems biology approach employed here serves as a broadly applicable framework for deciphering complex stress tolerance mechanisms in other industrially relevant microbes or under different challenging conditions. Combining such ALE-driven insights with rational synthetic biology offers a promising strategy for rapidly engineering microbes with enhanced traits, accelerating the transition toward a sustainable, methanol-based bioeconomy. These insights not only advance our understanding of methanol adaptation but also identify mutational targets and transcriptional responses that can inform rational engineering of methylotrophic platforms for the sustainable bioproduction of C1-derived value-added chemicals such as organic acids and bioplastics.

## Supplementary Information

Supplementary Material 1.

Supplementary Material 2.

Supplementary Material 3.

## Data Availability

The whole genome sequencing data generated in this study have been submitted to the NCBI SRA database (https://www.ncbi.nlm.nih.gov/bioproject/) under accession number PRJNA1304672, reviewer's accession link: https://dataview.ncbi.nlm.nih.gov/object/PRJNA1304672?reviewer=7sfve5hm3a9oli56lge169e8d9. The transcriptomics data discussed in this publication were deposited in the NCBI Gene Expression Omnibus (GEO) database under accession number GSE297705 (secure token: gxofosictxezrer) and are available at the following URL: https://www.ncbi.nlm.nih.gov/geo/query/acc.cgi?acc=GSE297705. Any additional information required to reanalyze the data reported in this paper is available from the lead contact upon request.
