# Supplementary material for: Integrated genomic and transcriptomic Insights into methanol tolerance mechanisms in Methylobacterium extorquens AM1, identifying key targets for strain engineering
Source: J Biol Eng. 2025 Dec 8;20:2. doi: 10.1186/s13036-025-00557-1 (PMC12797532; doi:10.1186/s13036-025-00557-1)
Supplement: Supplementary file 1 — Supplementary Material 1. [file 13036_2025_557_MOESM1_ESM.docx]

**Integrated Genomic and Transcriptomic Insights into Methanol Tolerance Mechanisms in *Methylorubrum extorquens* AM1 for C1-Based Bioproduction**

**Gyu Min Lee^a^, Khoi Nhat Pham^a^, Ina Bang^a^, Seyoung Ko^a, *^, Donghyuk Kim^a, *^**

^a^ School of Energy and Chemical Engineering, Ulsan National Institute of Science and Technology (UNIST), Ulsan 44919, Republic of Korea

*** Correspondence:**

E-mail address: kyumin1012@unist.ac.kr (Gyu Min Lee), khoi91hp@gmail.com (Khoi Nhat Pham), godlz0360@unist.ac.kr (Ina Bang), sierrayk@gmail.com (Seyoung Ko), dkim@unist.ac.kr (Donghyuk Kim),

Tel: +82-52-217-2945 (Donghyuk Kim), +82-52-217-3553 (Seyoung Ko)

Fax: +82-52-217-3009 Donghyuk Kim, Seyoung Ko)

**Additional files**

**Additional File 1: PDB file of the predicted MetY structure.**

The provided file (AF3_metY(MEXAM1_RS11880)_homotetramer.pdb) contains the atomic coordinates in Protein Data Bank (PDB) format for the homotetrameric structure of the *M. extorquens* AM1 MetY protein. This structure was predicted using AlphaFold3 and is the basis for the visualizations presented in Supplementary Figure S3.

**Supplementary Figures**

**
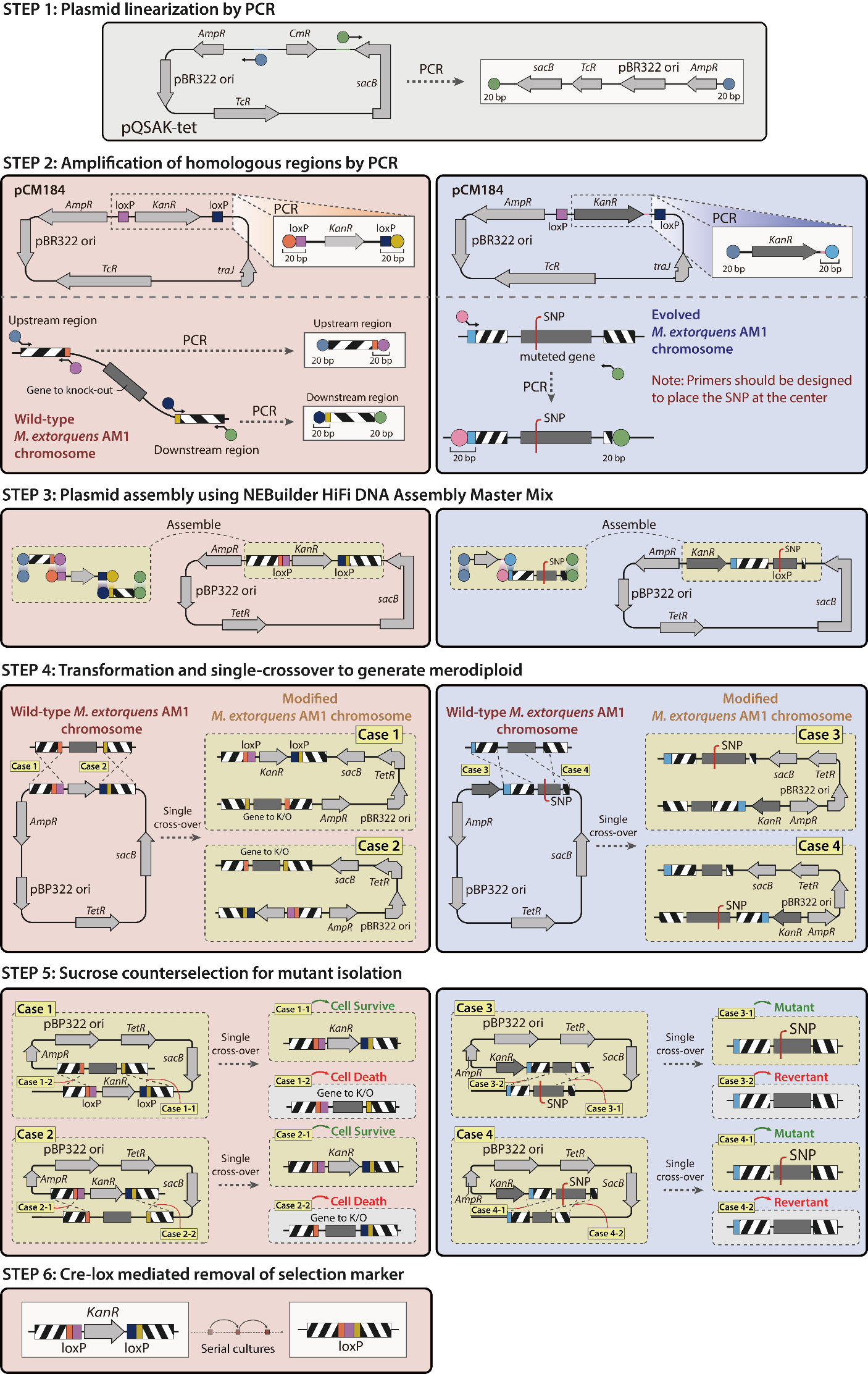
**

**Supplementary Figure 1. Stepwise workflow for gene knock-out and SNP introduction in *M. extorquens* AM1.** The knockout strategy for the *kefB* gene and the SNP introduction strategy for the *metY* gene are outlined in red and blue boxes, respectively. Step 1, which is shared by both strategies, is highlighted in gray, while key features and critical elements within each step are highlighted in yellow. Steps 2-5 correspond to the same experimental stages for both strategies, but the detailed procedures differ. Cases of single cross-over events that result in cell death are indicated in light gray. The knockout strategy employs a cre-lox system and includes an additional Step 6 to remove the selection marker.

**
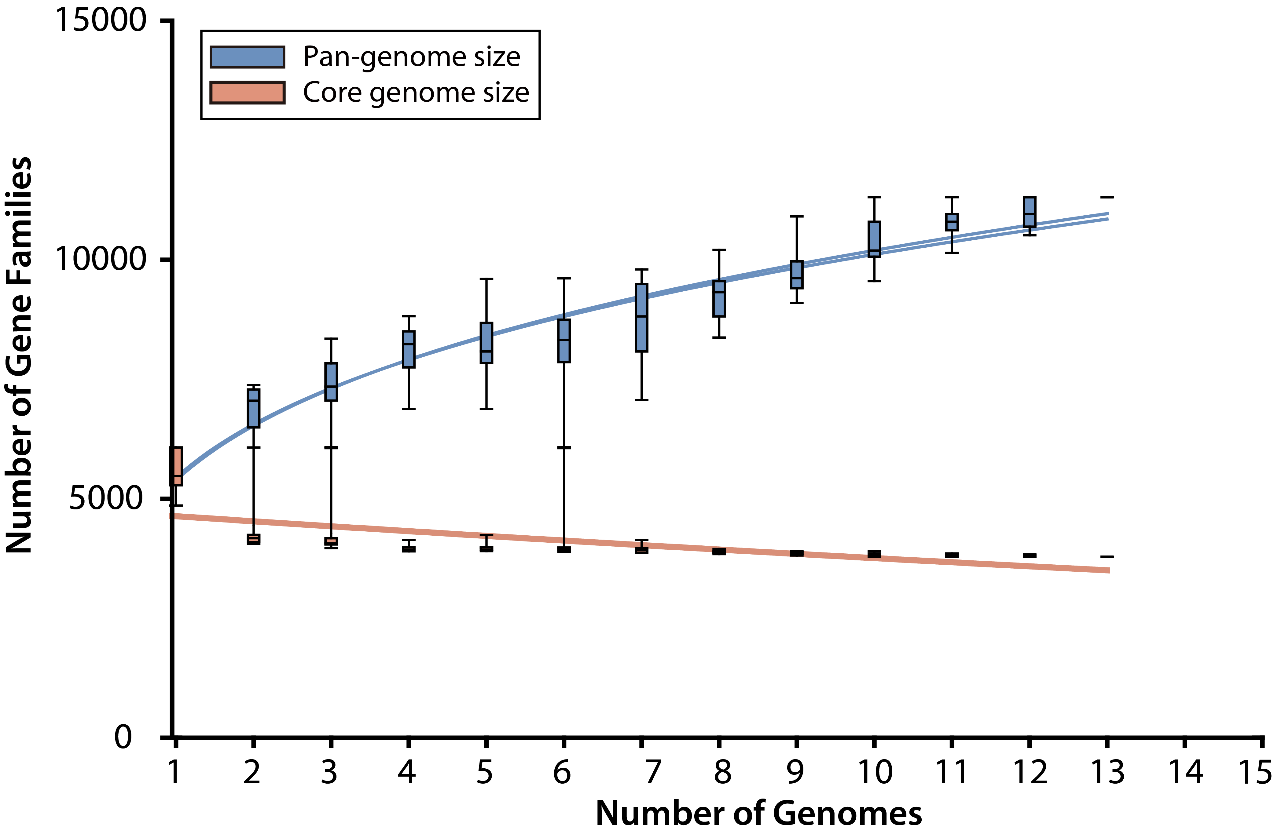
**

**Supplementary Figure 2. Pan-core plot of *M. extorquens* strains.** The number of gene families in the pan-genome and core genome of eight *M. extorquens* strains and five methanol-adapted strains is plotted against the number of genomes analyzed. The pan-genome size (blue) increased with the number of genomes included in the analysis, representing the cumulative set of unique gene families across the strains. The core genome size (orange) decreased as more genomes were analyzed, representing the set of gene families conserved across all strains. Boxplots indicate the variation in gene family counts due to different genome combinations at each genome count, and error bars represent the standard deviation.

**
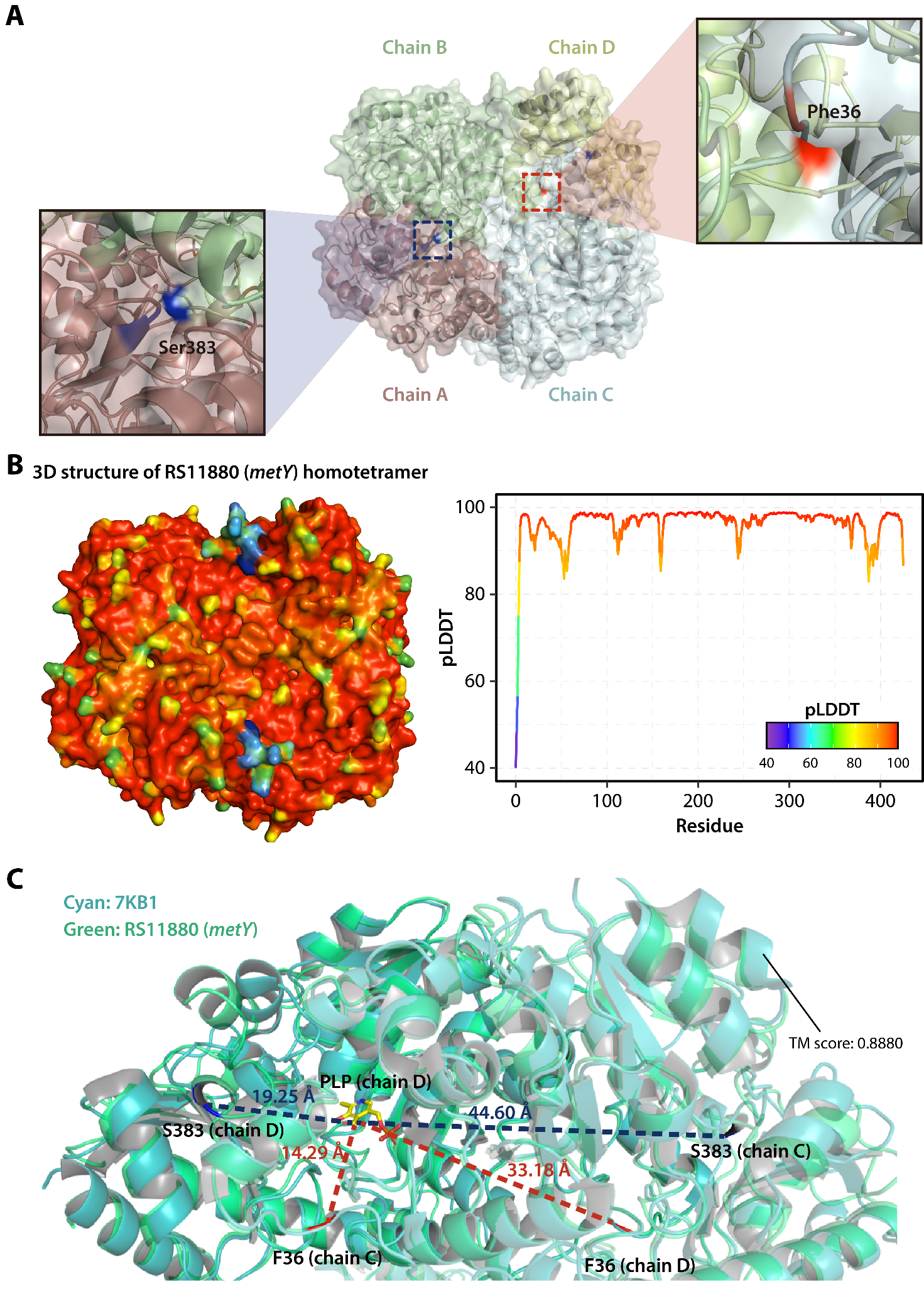
**

**Supplementary Figure 3. Structural modeling of the wild-type MetY homotetramer and localization of residues affected by adaptive mutations.** (A) Predicted 3D structure of the MetY homotetramer in *M. extorquens* AM1. The AlphaFold3- predicted 3D structure of the wild-type MetY homotetramer is depicted, with individual subunits distinguished by color and rendered in transparent surface. The native residues F36 and S383, which are the sites of adaptive mutations F36L and S383L identified in evolved strains, are highlighted to indicate their spatial positions. The right inset highlights the position of F36 within the wild-type structure, and the left inset highlights the position of S383. (B) Per-residue confidence of the predicted MetY structure. The homotetramer structure is colored according to the predicted Local Distance Difference Test (pLDDT) score provided by AlphaFold3. The color spectrum follows a rainbow palette, ranging from red (highest confidence) to violet (lowest confidence), providing a visual assessment of the prediction's reliability. (C) Structural alignment of MetY with the homologous protein from *Thermotoga maritima* (PDB: 7KB1). The wild-type *M. extorquens* MetY structure was superposed onto the experimentally resolved structure of *T. maritima* MetY containing a bound PLP cofactor. The positions of F36 and S383 are marked to illustrate their distances (>14 Å) from the PLP-binding site, indicating that these residues are structurally distant from the active site. The structure represents the wild-type sequence; mutant residues were not modeled.


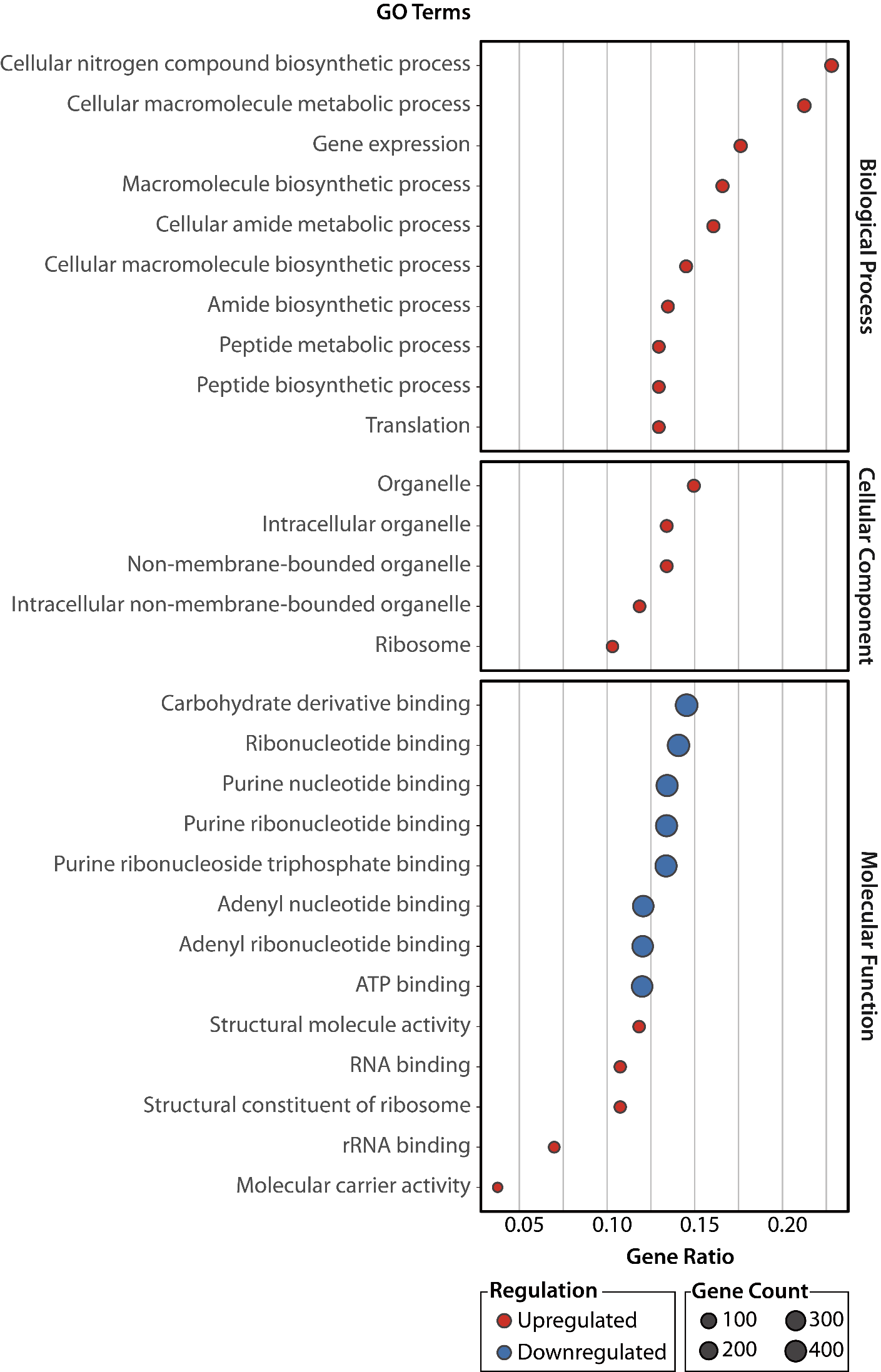


**Supplementary Figure 4. Gene Ontology (GO) enrichment analysis of DEGs.** GO terms are categorized into biological process (BP), cellular component (CC), and molecular function (MF). Upregulated and downregulated GO terms are indicated in red and blue, respectively. The dot size represents the number of genes associated with each GO term, with the gene ratio (x-axis) indicating the proportion of DEGs within the input gene set.
